# Anatomy of hypothalamic and diencephalic nuclei involved in seasonal fertility regulation in ewes

**DOI:** 10.3389/fvets.2023.1101024

**Published:** 2023-02-16

**Authors:** Miguel Merchán, Rafael Coveñas, Ignacio Plaza, José Alfonso Abecia, Carlos Palacios

**Affiliations:** ^1^Animal Production Area, Department of Construction and Agronomy, Faculty of Agricultural and Environmental Sciences, University of Salamanca, Salamanca, Spain; ^2^Laboratory of Neuroanatomy of the Peptidergic Systems, Institute for Neuroscience of Castilla y León (INCYL), University of Salamanca, Salamanca, Spain; ^3^Recognized Research Group - Molecular Bases of Development (Grupo de Investigación Reconocido - Bases Moleculares del Desarrollo - GIR-BMD), University of Salamanca, Salamanca, Spain; ^4^Auditory Neuroplasticity Laboratory, Institute for Neuroscience of Castilla y León (INCYL), University of Salamanca, Salamanca, Spain; ^5^Environmental Science Institute (IUCA), University of Zaragoza, Zaragoza, Spain

**Keywords:** calbindin, calretinin, parvalbumin, GFAP, IBA1, Nissl staining

## Abstract

In this study, we describe in detail the anatomy of nuclei involved in seasonal fertility regulation (SFR) in ewes. For this purpose, the intergeniculate leaflet of the visual thalamus, the caudal hypothalamic arcuate nucleus, and suprachiasmatic, paraventricular and supraoptic nuclei of the rostral hypothalamus were morphometrically and qualitatively analyzed in Nissl-stained serial sections, in the three anatomical planes. In addition, data were collected on calcium-binding proteins and cell phenotypes after immunostaining alternate serial sections for calretinin, parvalbumin and calbindin. For a complete neuroanatomical study, glial architecture was assessed by immunostaining and analyzing alternate sections for glial fibrillary acidic protein (GFAP) and ionized calcium-binding adapter molecule 1 (IBA1). The results showed a strong microglial and astroglia reaction around the hypothalamic nuclei of interest and around the whole 3^rd^ ventricle of the ewe brain. Moreover, we correlated cytoarchitectonic coordinates of panoramic serial sections with their macroscopic localization and extension in midline sagittal-sectioned whole brain to provide guidelines for microdissecting nuclei involved in SFR.

## 1. Introduction

Sheep are ruminants and seasonal breeders. As a result, sheep farming is misaligned with market demands (e.g., continuous production throughout the year), and the income derived from these seasonal animals is lower than that derived from non-seasonal animals (1). For this reason, one of the most important goals for seasonal animal husbandry (e.g., sheep) is to achieve continuous production throughout the year by shortening the periods between lambing, thereby improving production rates (2, 3).

Seasonality, an adaptive process whereby animals adapt their physiological functions (e.g., reproduction) to the environmental conditions (4), has been studied in several species ranging from invertebrates to vertebrates (5–7). In particular, research has focused on understanding the neurological basis of seasonal breeding among farm animals (e.g., sheep and goats) (8–10). Thus, structural plasticity changes, cell proliferation and migration in the sheep hypothalamus indicate that seasonal light changes induce a deep neural reorganization (11–13). Furthermore, recent studies have shown that polymorphisms in genes encoding melatonin 1A and 1B receptors (*MTNR1A* and *MTNR1B*, respectively) are involved in reproductive seasonality (14–17). Concurrently, anatomical and physiological studies have implicated melatonin in the control of seasonal breeding or estrous cyclicity (18, 19). Based on these findings, attempts have been made to regulate the circadian cycle of sheep by changing their natural photoperiod in combination with melatonin implants and hormone treatments (20–22).

In immunohistochemical studies, kisspeptin, neurokinin A and dynorphin B, called KNDy neurons, and Gonadotropin Releasing hormone (GnRH) neurons have also been identified in the sheep hypothalamus, highlighting their key role in the endocrine regulation of seasonal breeding (8, 23–25). However, analyzing interactions and the complex neuronal machinery involved in the hypothalamic regulation of seasonality requires understanding its neuroanatomical underpinnings. The main nuclei involved in circadian rhythm regulation in rodents and non-human primates are the hypothalamic suprachiasmatic nucleus (SCh) and the diencephalic intergeniculate leaflet (IGL) nucleus of the thalamus (26). Other hypothalamic nuclei also involved in seasonality include the supraoptic (SO), paraventricular (PVN) and arcuate (ARC) nuclei (27, 28). With slight differences in morphology and spacial distribution, these nuclei are present in all higher mammals, including sheep (11, 26, 29–31). Full neuroanatomical and neurochemical studies of hypothalamic nuclei involved in seasonal regulation (e.g., circadian rhythms and seasonal changes in GnRH regulation) must be conducted to gain further insights into the neural basis of fertility regulation in sheep (24, 32, 33).

A few anatomical atlases of the ruminant brain (e.g., sheep, goat, and alpaca) have been previously published (34–37). These studies have used a wide range of methods, including gross anatomy, high-resolution 3D Magnetic Resonance Imaging, and immunohistochemistry or Nissl-stained sections. A highly detailed general description of sheep diencephalic nuclei was reported in a doctoral dissertation dating back to 1976, using coronal thionine-stained seriated sections (38) however it was not interpreted in the context of the neuromeric model. Thus, further research should be conducted to analyze in detail the sheep neuroanatomical structures involved in seasonal breeding.

In this article, we describe the anatomical analysis of SFR nuclei using Nissl-stained serial sections and immunocytochemistry for CBPs. For this purpose, we selected three CBPs, namely Cr, Cb and PV, which have been widely used as markers for cytoarchitectural analysis and for neuronal phenotyping in different vertebrate species (39, 40).

Considering the above, the present study aimed at, (1) determining the morphological features and relative position (coordinates) of seasonal fertility regulation (SFR) nuclei in the ewe diencephalon and hypothalamus by analyzing Nissl-stained serial sections in the three anatomical planes based on high-resolution panoramic photographs, (2) studying the immunohistochemical calbindin (Cb), calretinin (Cr) and parvalbumin (Pv) profile of SFR nuclei, (3) analyzing the morphology, distribution, and cell density gradients (glial architecture) of microglial and astroglia cells in the ewe hypothalamus, and (4) generating a macroscopic map with the specific coordinates and extension of SFR nuclei to enable their accurate localization for microdissections.

## 2. Materials and methods

### 2.1. Experimental groups

Six adults female Churra sheep (age = 16.60 ± 0.25 months); mean live weight [(LW) = 70 ± 6 kg] housed under natural light, temperature, and humidity conditions, fed *ad libitum* and provided with unrestricted access to clean drinking water, were used in this study over farm natural conditions. The experiments were performed in accordance with Spanish (RD 53/2013) and European (63/2010/EU) directives, and animal maintenance and care met all requirements of the agreement on the use of animals in scientific experimentation in Spain (Confederation of Scientific Societies of Spain - COSCE). Accordingly, the animals were sacrificed at the slaughterhouse MACRISA, in Medina de Rioseco, Valladolid, Spain, in April 2021, by neck cutting, severing the carotid and jugular veins, without prior drug administration. Heparinized 5-ml tubes were used to collect blood samples by jugular venipuncture; samples were immediately centrifugated at 3000 × g for 20 min, and plasma was stored at −20°C. Hormonal levels of progesterone was assayed by radioimmunoassay (RIA). All samples were run in a single assay. In all specimens, the progesterone levels were homogeneous, ranging from 15 to 20 mg/l.

### 2.2. Animal samples

After removing the skin, a horizontal cut was made in the skull from the external occipital protuberance to the upper edges of the orbits using an ultra-fast surgical saw with a 5 cm diameter radial disc. Using this approach, the dorsal superficial area of the cortex was cleanly cut, thus showing in depth the ventricles in the middle line maintaining intact the diencephalic area. This procedure provided access to the ventricles for a faster and more effective fixation. After tilting the skull backwards with a spatula and a pair of tweezers, the brain was gently pushed to ventrally cut the optic nerves to rostrally free the brain and to reveal and cut the pituitary stem. Caudally cranial nerves and meningeal flanges were severed. As a result, the brains shed and fell off under their own weight. The nerves were cut with a vise, and the meninges were removed from the pituitary stalk to facilitate infiltration of the fixative solution. The time elapsed from euthanasia to brain and pituitary fixation was approximately 5 min.

### 2.3. Histology

#### 2.3.1. Fixation and sectioning

Each brain was placed in a biosafety container with 1500 ml of 4% paraformaldehyde in 0.1 M phosphate buffer, pH 7.4 at 4°C. After 48 h, to obtain standardized anatomical planes, the brains were coronally cut from the optic chiasm to 1 cm caudally to the mamillary body, yielding a squared block containing almost the entire diencephalon. Block tissues were fixed for one more week after renewing the fixation solution every 48 h and then immersed in solutions with increasing sucrose concentrations (from 20 to 100%) until they sank. Subsequently, diencephalic blocks were carved to obtain standardized cutting planes and sectioned serially in the coronal, sagittal or horizontal plane at 40 μm using a freezing microtome. For coronal sections, whole diencephalons (left and right sides: four animals) were used, whereas the rest of the diencephalons studied (two animals) were cut through the midline, using each side to prepare sagittal or horizontal sections. In addition, 3600 Nissl-stained coronal histological sections were obtained from the four diencephalons, whereas 900 sagittal and 900 horizontal Nissl-stained histological sections were obtained from the other two diencephalons. In total, 1080 coronal histological sections were used for calcium-binding proteins (CBPs) immunohistochemistry and 720 coronal histological sections for ionized calcium binding adaptor molecule 1 (IBA1) and glial fibrillary acid protein (GFAP) immunohistochemistry. After fixation, a 3% retraction occurred in the diencephalic blocks. After sectioning, in Nissl-stained sections, the thickness retraction was 4 microns, as calculated by three experienced microscopists using a x100 Leica plan apochromatic objective. Thus, histological sections stained with Nissl had an estimated final thickness of 36 μm.

#### 2.3.2. Nissl staining

**Figure 1:**
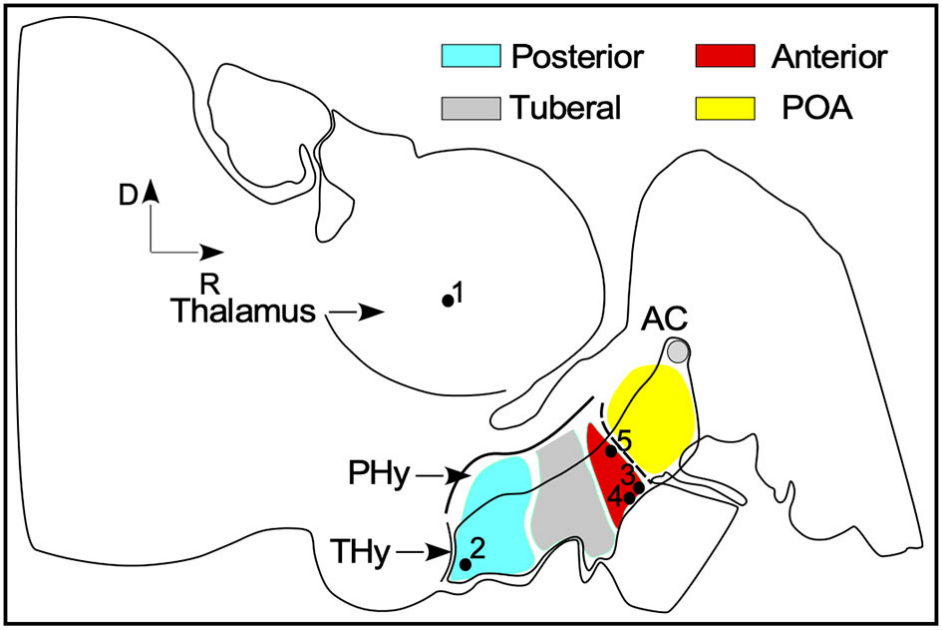
General scheme of the ewe brain in the sagittal plane inspirated in the neuromeric model for brain organization. SFR nuclei rough position are labeled by black dots: (1) IGL - thalamus (2) ARC- posterior blue area (3) SCh and (4) SO nuclei (5) PVN in the anterior red area. AC, anterior commissure; PHy, peduncular hypothalamus; Thy, terminal hypothalamus; POA, preoptic area.

Sections were stained by immersion in 1% cresyl violet (C5042, Sigma-Aldrich, Merck KGaA, Darmstadt, Germany) for 10 min at pH 3.0 and in 96% alcohol + acetic acid for staining differentiation. Lastly, the sections were dehydrated in increasing alcohol concentrations, from 50 to 100%, followed by clearing in xylene (3 × 3 min), mounting and cover slipping. The nomenclature used in this paper followed that reported in the Paxinos' atlas of the mouse brain (41). SFR nuclei of this atlas were clearly recognized in our material. In addition, for an appropriate localization of SFR nuclei in the ewe brain, we followed the guidelines based on a recently updated neuromeric model developed in rodents (42) (Figure 1).

#### 2.3.3. Immunostaining

Alternate coronal serial sections were stained for calbindin, calretinin, parvalbumin, IBA1 and GFAP (for details about the antibodies used, see Table 1). Free-floating sections were sequentially washed with 0.05 M tris buffer saline, pH 7.6, followed by endogenous peroxidase inhibition by incubation in 10% methanol + 3% H_2_O_2_ in 0.1 M PB for 10 min. Subsequently, the sections were washed in 0.1 M PB and 0.05 M TBS-Tx, pH 8.0, 0.3% Triton X-100 (T9284 Sigma, St. Louis, MO, USA; TBS-Tx) and incubated with the corresponding primary antiserum (Table 1), for 48 h at 4°C. Non-specific labeling was blocked using fetal calf serum (10%). After washing three times in TBS-Tx, for 15 min, all sections were incubated with an anti-rabbit biotinylated secondary antibody (biotinylated anti-rabbit IgG H+L, BA-1000; Vector, Burlingame, CA, USA) or with an anti-mouse biotinylated secondary antibody (biotinylated anti rabbit IgG H+L, BA-2000; Vector, Burlingame, CA, USA) at a 1:200 dilution in TBS-Tx for 120 min at room temperature. The sections were then washed with TBS-Tx and incubated for 180 min in avidin/biotin–peroxidase (ABC complex, Vectastain Standard ABC kit PK-4000; Vector, Burlingame, CA, USA) and further washed with TBS-Tx, followed by Tris-HCl, pH 8.0. Lastly, the sections were incubated in 3,3-diaminobenzidine tetrahydrochloride (DAB; D-9015; Sigma-Aldrich, St. Louis, MO, USA) with 0.006% H_2_O_2_ to visualize the peroxidase reaction. Negative controls, processed without the corresponding primary antibody, were performed to confirm immunostaining specificity; in all cases, the results showed the specificity of the primary antibodies used in this research. Calcium-binding protein cell phenotype analysis across a high number of representative mammalian species showed that the types of positive neurons generally coincided among species. Previous analysis indicated that these proteins have a highly phylogenetically conserved molecular structure in the brain in general and in the cerebellum in particular (30, 43, 44). Thus, superior colliculus and cerebellum (Supplementary Figure 1) have been analyzed as positive controls, showing immunostaining properties similar to those reported for humans and other high mammals, such as the common marmoset (29). Antibodies used by us are not commercially validated for sheep. However, previous literature reported the use of these antibodies in sheep (45–48). In the case of the antibody against IBA1 used in our paper, we have performed a BLASTp analysis of the 16 amino acid epitope (NPTGPPAKKAISELPC) used as immunogenic protein against the sheep genome, and we have obtained 92.86% homology: Results for BLASTP against Sheep Oar_rambouillet_v1.0 [Proteins (Ensembl)] - Ovis_aries_rambouillet - Ensembl genome browser 108.

**Table 1:** Characteristics of the antibodies and dilutions used in this study.

**Antigen**	**Dilution**	**Inmunogen**	**Description**
Calbindin	SWANT CB38a 1:5000	Recombinant rat calbindin D-28k (CB)	Polyclonal rabbit Swant Cat# CB38, RRID: AB_10000340
Calretinin	SWANT SW/7697 1:2000	Recombinant human calretinin containing a 6-his tag at the N-terminal	Polyclonal rabbit Swant Cat# CR 7697, RRID: AB_2619710
Parvalbumin	Sigma P-3088 1:1000	Frog muscle parvalbumin.	Monoclonal mouse Sigma-Aldrich Cat# P3088, RRID: AB_477329
GFAP	Sigma G-6171 1:500	Purified GFAP from pig spinal cord	Monoclonal mouse Sigma-Aldrich Cat # G6171, RRID: AB_1840893
IBA1	Wako 019-19741 1:1000	C-terminus of Iba l' (NPTGPPAKKAISELPC')	Polyclonal rabbit, Wako Cat # 019-19741, RRID: AB_839504

#### 2.3.4. Image analysis

Panoramic mosaics of entire sections of the rostral half of the brain were captured at selected interaural coordinates. Digital photomicrographs (mosaics) were taken under a Leica DMRB microscope with X10 objectives (Leica Plan Apo), assembled using the “Virtual slice” module of Neurolucida 8.0 (MBF-Bioscience, Williston, Vermont, USA), and adjusting the microscope illumination source before each image capture. To measure the area and perimeter of the cells, photomicrographs of Nissl-stained sections were processed in ImageJ 2.0 software. Using the automatic method of analysis by density threshold segmentation, a detection range was applied to select the appropriate outlines of cell bodies, which were visually inspected to avoid counting superimposed or confluent groups of cells. To enhance photographs by pseudo-color transformation, improving the delimitation of Nissl-stained panoramic neuronal nuclei, we used different Image J software plugins (ICA 3, Spectrum, and 16-color tables). To numerically assess differences in the density of coronal sections immunostained with IBA1, mean gray levels were analyzed (ImageJ program) in selected frames taken on the horizontal axis at different distances from the 3^rd^ ventricle.

## 3. Results

### 3.1. Serial Nissl staining sections of the diencephalic and hypothalamic SFR nuclei

**Figure 2:**
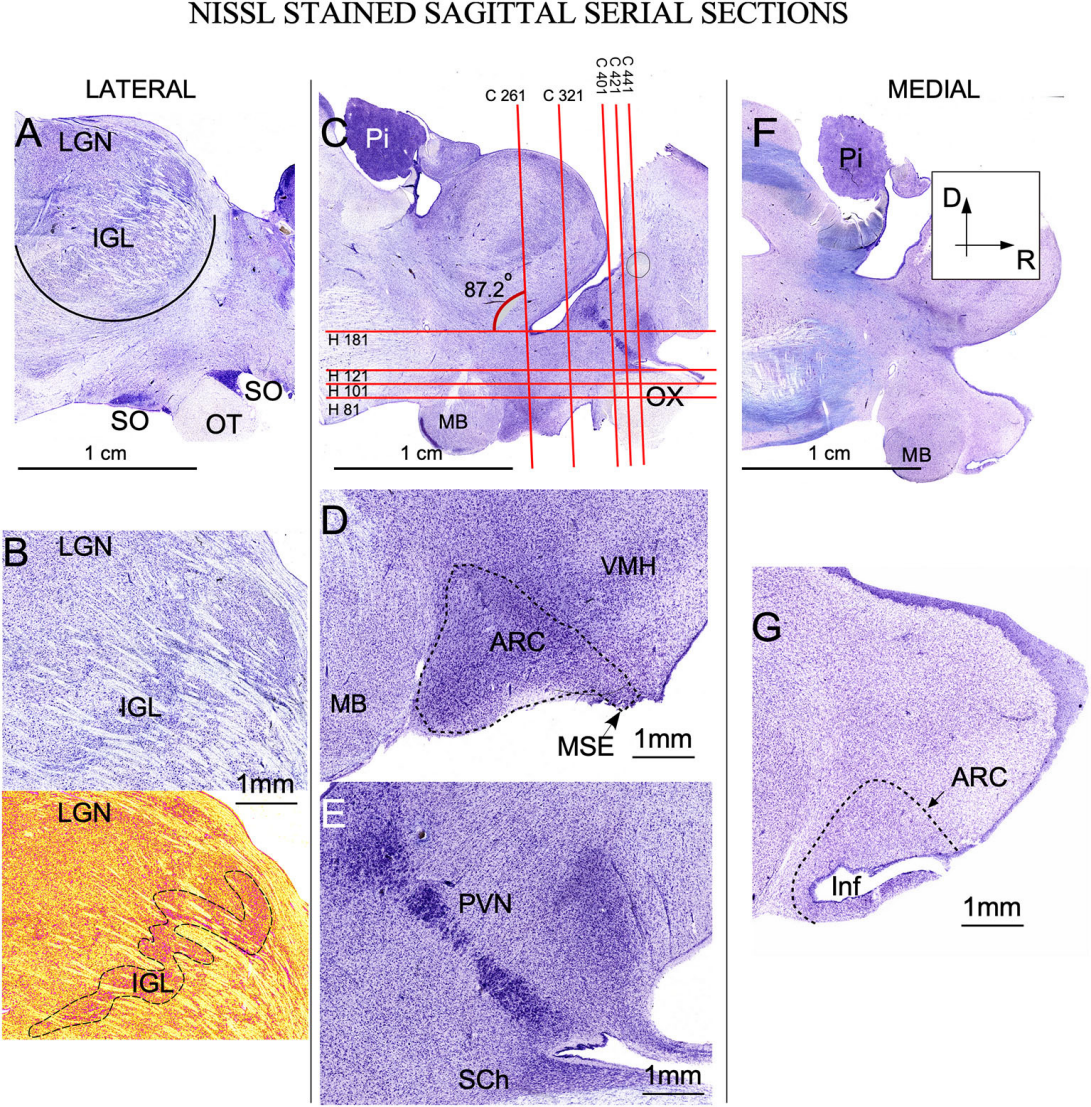
Nissl-stained sagittal serial sections of the ewe diencephalon. **(A)** Panoramic view of a lateral longitudinal section showing the intergeniculate leaflet (IGL), crossing ventro-dorsally the lateral geniculate nucleus (LGN). Ventrally, the optic tract (OT) in close apposition with the supraoptic nucleus (SO). **(B)** Higher magnification of IGL; in the bottom, a pseudocolor image allows us to distinguish the meandering trajectory of IGL (dotted line). **(C)** More medial section showing all nuclei of interest. Red horizontal and vertical lines defined the level of sectioning corresponding to coronal (C 261 …) or horizontal (H 81 …) serial sections shown in Figures 3, 4. **(D)** Higher magnification showing the arcuate nucleus (ARC) distributed from the (MB) mamillary body to the stalk of the median eminence (MSE). **(E)** Detail of the rostral area in which the periventricular nucleus (PVN) can be distinguish as a strongly Nissl-stained band of cells close to the 3^rd^ ventricle. Ventrally suprachiasmatic nucleus (SCh) is observed between the optic tract and the lateral recess. **(F)** Lateral most panoramic section. **(G)** Detail showing the infundibulum (Inf). Pi, Pineal gland; VMH, Ventromedial hypothalamus nucleus. **(A, C, F)** same magnification.

**Figure 3:**
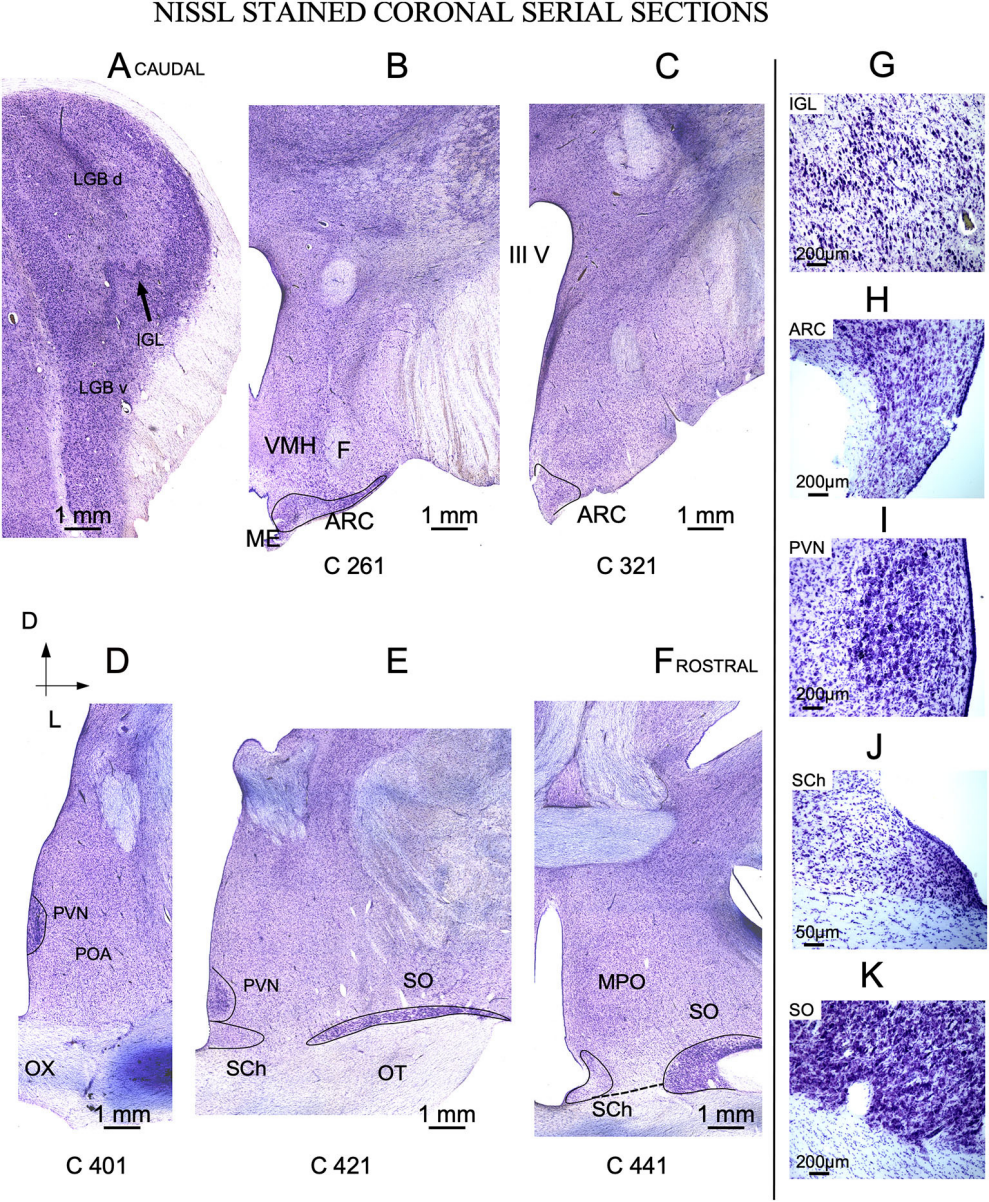
Nissl-stained coronal serial sections. **(A)** Caudal section showing the dorsal (LGB d) and ventral (LGB v) divisions of the lateral geniculate body separated by the intergeniculate leaflet (IGL). **(B, C)** Arcuate nucleus (ARC) located I the caudal hypothalamic red area in Figure 1. **(D–F)** Three representative sections from the rostral hypothalamus (blue area in Figure 1). The labels in the bottom of each image (C 261 to C 441) match the level of sectioning corresponding with vertical red lines of Figure 2. **(G–K)** Details of Nissl-stained cells from all nuclei of interest. OX, Optic chiasm; MPO, Medial preoptic area; ME, Medial eminence; VMH, ventro-medial nucleus; IIIV, third ventricle- **(A–F)** same scale bar.

**Figure 4:**
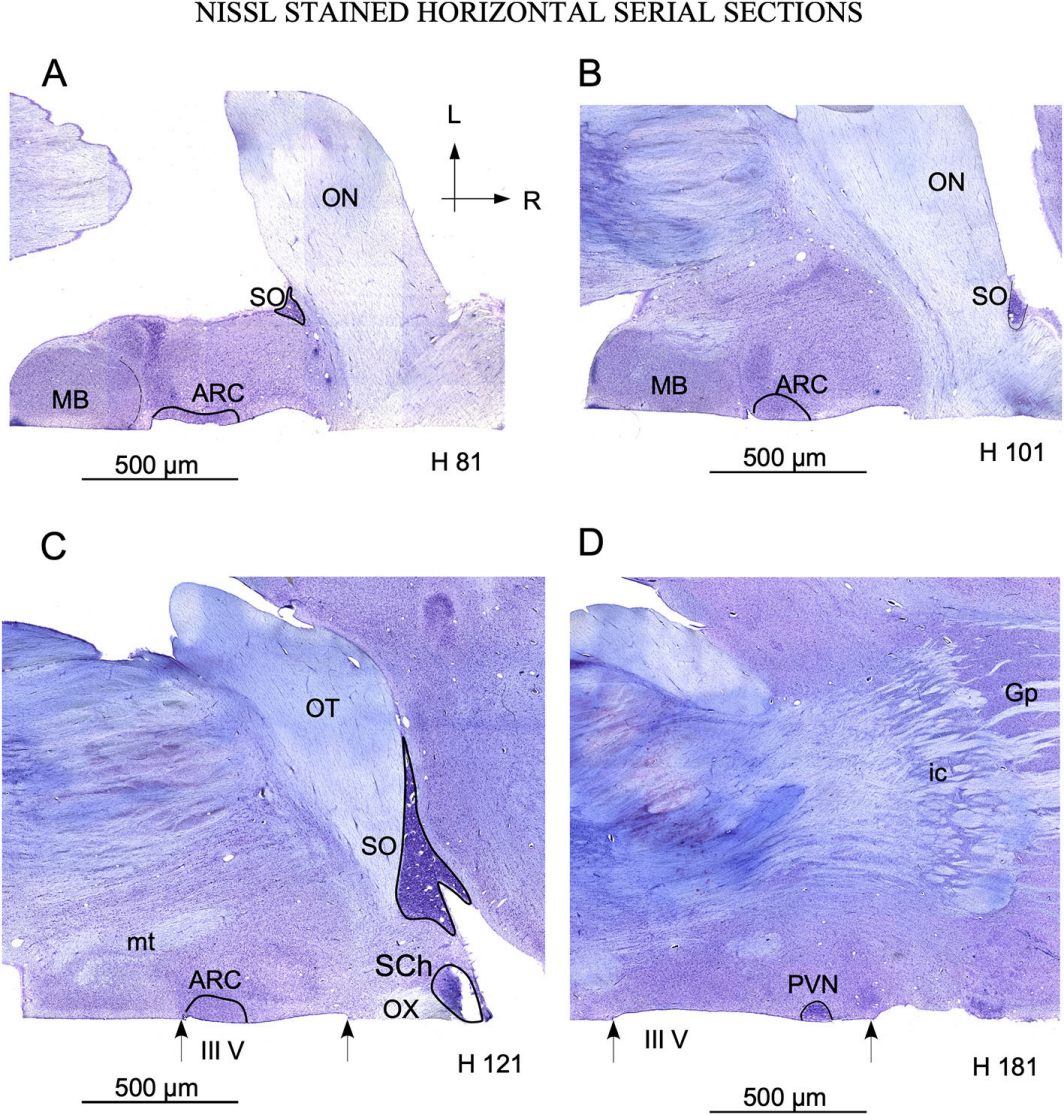
Serial sections in the horizontal plane ordered from ventral **(A)** to dorsal **(C)**. **(A–C)** show the localization of arcuate nucleus (ARC) adjacent to the mamillary body (MB), bordering the 3^rd^ ventricle (III V) delimited by arrows. The SO nucleus is located laterally in the ventral sections. **(A, B)** and medially in dorsal sections **(C, D)**, as a consequence of its distribution around the optic tract. Note the localization of paraventricular nucleus (PVN) with respect to the 3^rd^ ventricle in **(D)**. The labels in the bottom of each image (H 81 to H 181) match the level of sectioning corresponding with horizontal red lines of Figure 2. Ic, internal capsule; GP, Globus pallidus; mt, mammillothalamic tract; OT, Optic tract. **(A–D)** same magnification.

To set cytoarchitectonic boundaries in panoramic high-resolution images, nuclei were delimited by exploring differences in cell density, cellular shape and size, and Nissl staining intensity. In this study, the preoptic area (POA), the anterior and the posterior areas of the terminal hypothalamus (THy), were analyzed to more easily describe the localization of SFR (Figure 1). This approach helped us to locate, in a single sagittal section, the four nuclei of interest, ARC in the posterior (Figure 1 dot 1), SCh and SO (Figure 1 dots 2, 3), in the anterior and PVN (Figure 1 dot 4) close to the POA. By contrast, in horizontal and coronal sections, the nuclei must be analyzed in several serial sections. To estimate the related position of coronal and horizontal sections with respect to the sagittal plane, the coordinates were calculated from the first rostral (for the coronal plane) and ventral (for the horizontal plane) sections from carved blocks of tissue (sectioning plane), by multiplying the order number in the series by the averaged thickness of the sections (36 μm) (Red lines in Figure 2C in correspondence with Figures 3, 4).

#### 3.1.1. Intergeniculate leaflet of the thalamus

The thalamic lateral geniculate nucleus (LGN) was large (approximately 1 cm in the main diameter), oval, and located laterally and dorsally in the brain (Figure 2A). Pseudo-color images allowed us to define IGL borders easily and clearly in Nissl-stained sections after color conversion (Figure 2B bottom). Thus, in both sagittal (Figure 2A) and coronal planes (Figures 3A, G), IGL was identified by the snake plot shape of a high-density and hyperchromatic band of cells. This morphological feature facilitated the identification of the thalamic IGL in the border between the dorsal and ventral LGN. However, due to the presence of separate clusters and to its convolved distribution, IGL was difficult to delimit in horizontal sections (not shown). IGL neuronal bodies were identified by their fusiform shape in the coronal plane and by their medium-to-large size (Area M: 649 μm^2^ – SD ± 346.6) (Figure 3G; Supplementary Table 1).

#### 3.1.2. Arcuate nucleus

In sagittal sections, from its rostral limit with the mammillary body, ARC appeared as a ribbon of cells, distributed, along the entire ventricle extension to the infundibulum (Inf) and the median stalk eminence (Figures 2D, G, dotted line). In the ventral THy, in the coronal plane, ARC wrapped around the 3^rd^ ventricle (Figures 3B, C) and was clearly distinguished for its strong Nissl staining, in all planes (Figures 2G, 4A–C). In horizontal sections, ARC was observed in the caudal third of the 3^rd^ ventricle (Figures 4A–C). In coronal sections, ARC was triangular, extended for approximately 3 mm dorsally, and showed strongly stained neurons (Figure 3H). Its neurons were medium to large (averaged area: 590 μm^2^ – SD ± 290.5), hyperchromatic, triangular and more densely packed in the ventral part (Supplementary Table 1).

#### 3.1.3. Paraventricular nucleus

PVN was easily delimited, in all planes, because its neurons were large and strongly Nissl stained (Figures 2E, 3D, E, 4D). PVN had a columnar shape in the sagittal plane (Figure 1E) and a round shape in the coronal (Figures 2D, E) and horizontal (Figure 3D) planes. PVN neurons were spherical and large (averaged area: 899 μm^2^ – SD ± 322.4) (Figure 2I; Supplementary Table 1).

#### 3.1.4. Suprachiasmatic nucleus

SCh was located medially and ventrally on the floor of the 3^rd^ ventricle, bordering the optic tract and chiasma. SCh was small, round, or oval at the horizontal plane (Figure 4C), and conical at the sagittal (Figure 2E) and coronal (Figures 3E–J) planes. According to Nissl staining, SCh can be subdivided in ventral and dorsal areas. SCh neurons had different shapes of neuronal somata, mostly with stellate and oval contours, with small or medium sizes (Averaged area: 161.9 μm^2^ – SD ± 181.2) (Figure 3J; Supplementary Table 1).

#### 3.1.5. Supraoptic nucleus

SO contained densely packed, large, and strongly Nissl-stained cell bodies. In the most rostral area, SO was elongated, slim, and oval in coronal sections (Figures 3E, F). Given its large size, this nucleus was observed along more than 20 rostral sections (Figures 3E, F). Because SO is close to the optic tract, at the horizontal plane, SO was identified at the lateral part of the most ventral horizontal sections (Figure 4A) and medially to the dorsal ones (Figure 4C). Thus, the optic tract and optic chiasm border the SO nucleus (Figures 2A, 3E, F, 4A–C), whose neurons are uniformly round or fusiform and large (Averaged area: 915 μm^2^ – SD ± 327.6) (Figure 2K; Supplementary Table 1).

### 3.2. Neuronal morphological features and CBPs immunostaining of the diencephalic and hypothalamic SFR nuclei

The neurons of SFR nuclei showed Cb and Cr, but not Pv, immunoreactivity. All CBPs immunocytochemistry results from serial sections in the coronal plane were compared with the corresponding Nissl-stained alternate sections in the coronal plane.

#### 3.2.1. Intergeniculate leaflet of the thalamus

**Figure 5:**
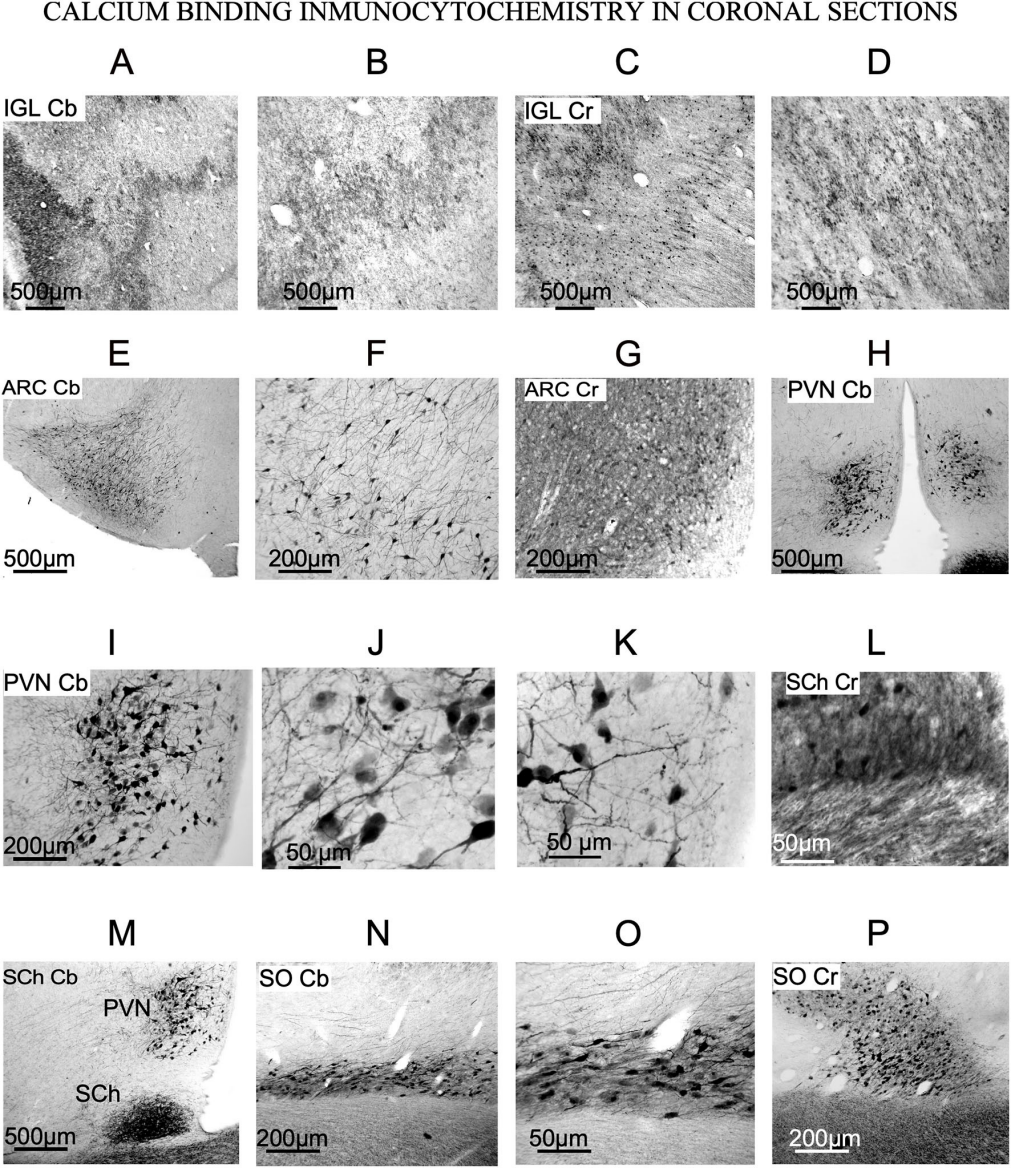
Calcium-binding protein immunoreactivity of the nuclei of interest (SFR). **(A–D)** Immunostaining of the IGL. Terminals are positive for calbindin and calretinin **(E–G)** Immunostaining of arcuate nucleus. Cb and Cr-stained neurons are evident. **(H–K)** Immunostaining of PVN. Large stellate neurons with thick dendrites are Cb positive. **(L, M)** Immunostaining of SCh. Cr and Cb positive fibers enter the nucleus from the optic chiasm [this photograph is a wider view of **(H)**]. **(N–P)** Immunostaining of SO, showing large Cb and Cr neurons oriented in the main axis of the nucleus.

Cb and Cr -stained sections showed, in IGL, restricted immunopositively terminal buttons, which made it possible to delineate the nucleus from LG subdivisions (Figures 5A–D). By immunoreactivity against Cr, dense oval cell bodies were observed (Figures 5C, D), as in Nissl-stained sections (Figure 3G). In Pv immunostained sections, the intense positive reaction along LGN blurred the nucleus, making it difficult to determine a specific reactivity in cell bodies (not shown).

#### 3.2.2. Arcuate nucleus

In ARC, stellates cells were immunoreactivity against Cb and Cr (Figures 5E–G). Those showing immunoreactivity to Cb were a specific group of stellate multipolar medium or small neurons with a large number of dendrites (Figures 5E, F). Cr-immunoreactive neurons were dispersed in the most ventral part, near the third ventricle (Figure 5G), such as small neurons observed in Nissl-stained sections (Figure 3H).

#### 3.2.3. Paraventricular nucleus

PVN neurons were not immunoreactive to Pv and Cr (data not shown) but were immunoreactive to Cb, which allowed us to observe large multipolar neurons with well-defined cell bodies and well-defined dendrites (Figures 5H–K). Similarly, in Nissl-stained sections neurons from PVN were large and round (Figure 3I).

#### 3.2.4. Suprachiasmatic nucleus

SCh contained Cb-immunoreactive neurons and showed positive fibers from the optic chiasm entering the nucleus, generating a dense terminal field (Figures 5L, M). After comparing immunoreactive against Cb with equivalent Nissl-stained sections, only a few of the Nissl-stained neurons (Figure 3J) were positive for this antibody (Figure 5L).

#### 3.2.5. Supraoptic nucleus

In our material, most SO neurons were immunopositive again Cb and/or Cr (Figures 5N–P). Immunopositively stained dendrites and cell bodies showed strong Cb and Cr staining (Figures 5N–P). Its medium-to-large neurons shows strongly immunopositive dendrites. Immunostained sections showed much smaller number of cells than the Nissl-stained ones (compare Figures 5N–P with Figure 2K).

### 3.3. Glial architecture IBA 1 and GFAP immunohistochemistry

**Figure 6:**
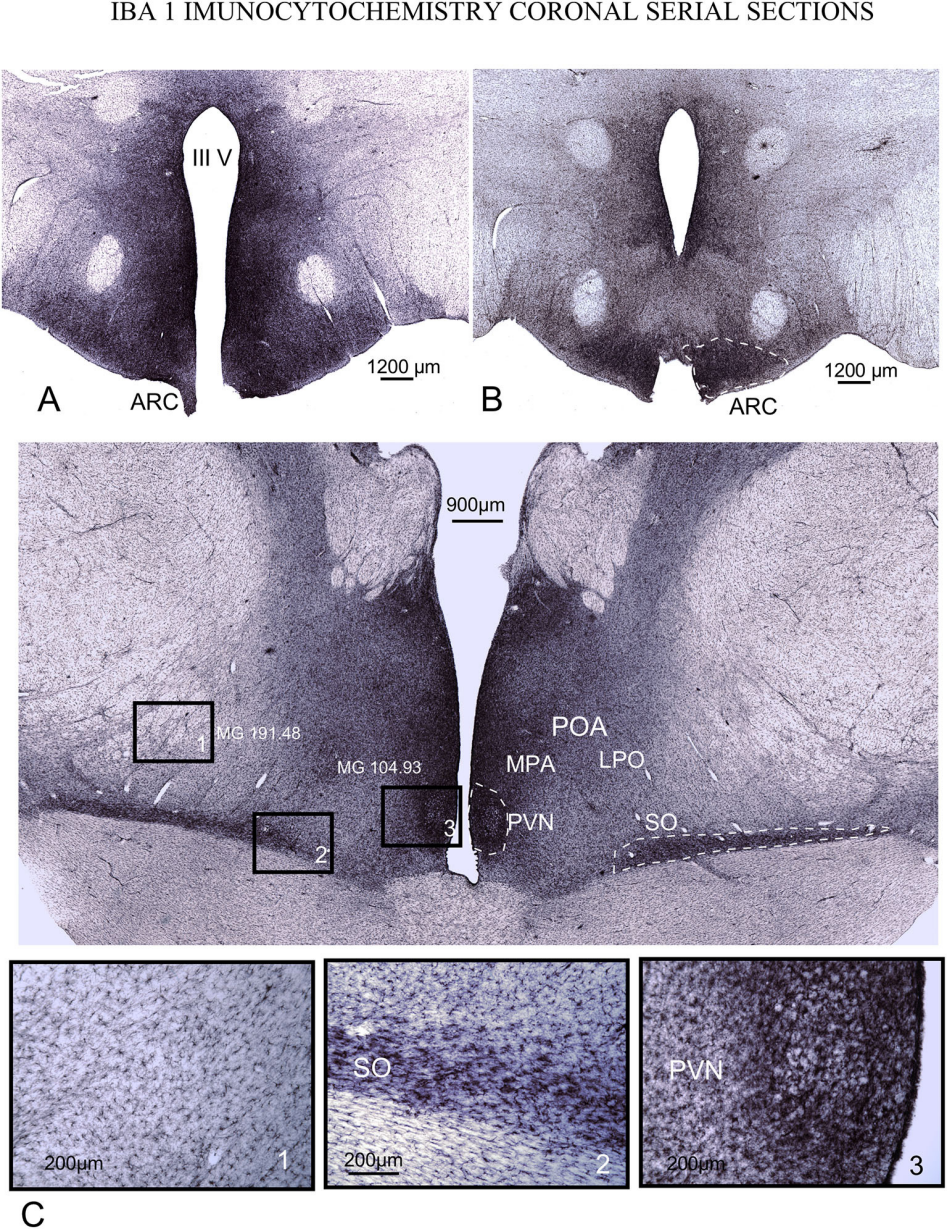
IBA1 immunoreactivity in serial coronal sections. **(A, B)** caudal ventromedial hypothalamus. Densest microglial reaction is found in the ARC (dotted line) and around the 3^rd^ ventricle. **(C)** POA. Details in squares from 1 to 3 (bottom of the figure) show that reactive microglial cells are highly dense and more concentrated around and inside PVN and SO nuclei. Note the intense staining around the 3^rd^ ventricle in all levels of sectioning. Mean gray level measured inside the squares (MG – mean gray value).

**Figure 7:**
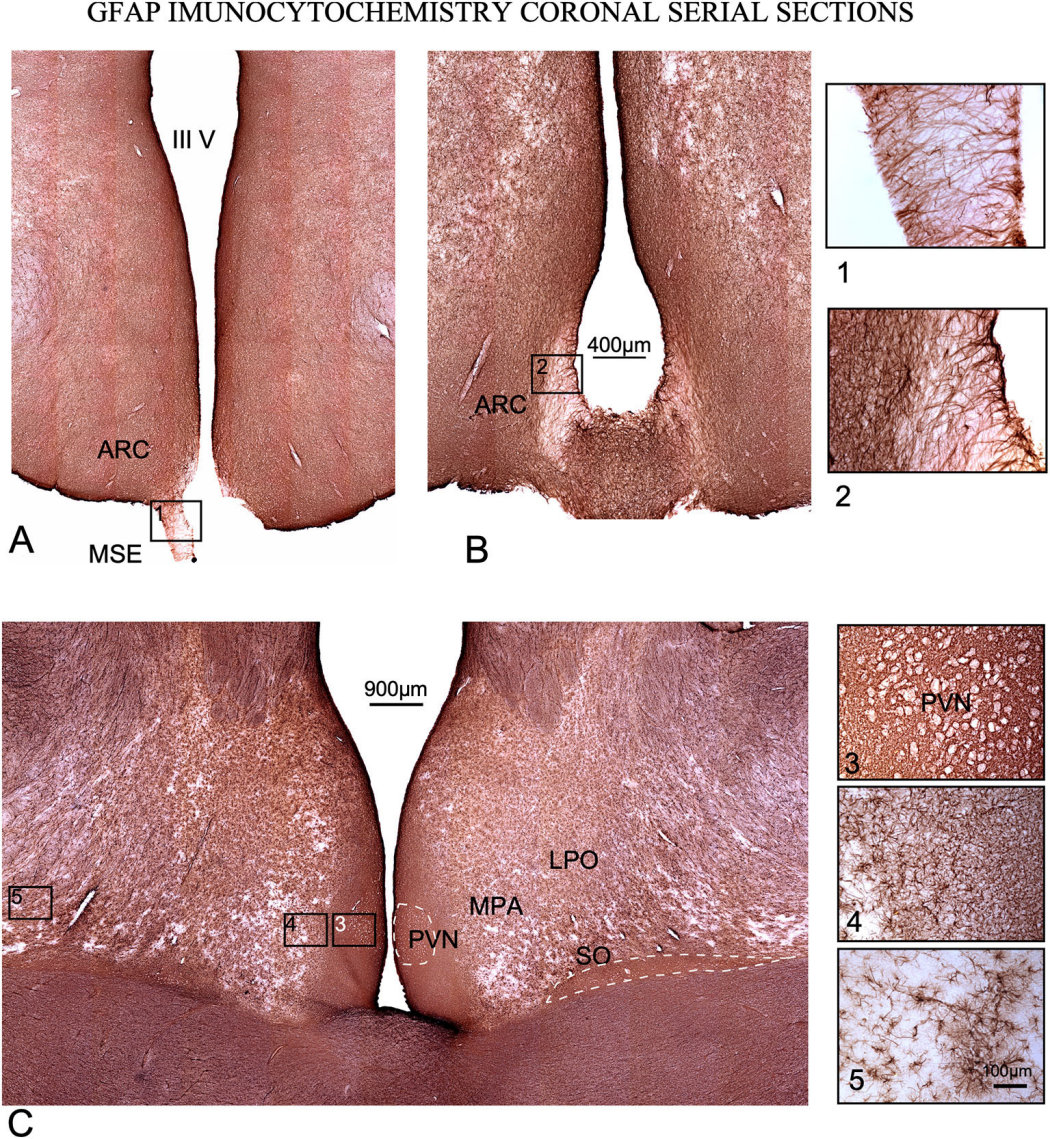
GFAP immunoreactivity in coronal serial sections. **(A, B)** caudal sections. Squares (1) and (2), tanycytes at the median stalk eminence (MSE). **(C)** Rostral section. Higher cell density is observed around the 3^rd^ ventricle in all levels of sectioning, and around and inside PVN and SO nuclei (dotted lines). Squares 3, 4, and 5 are shown at a higher magnification on the right of the figure. (3) Detail of immunoreactivity in PVN. Neurons encircled by astrocytes are shown as white contours. (4) Interstitial astroglia in the lateral preoptic area (LPO). (5) Non-reactive astrocytes far from the 3^rd^ ventricle.

No distinct features of astroglia and microglial cells were detected in IGL with respect to the remaining lateral geniculate body (not shown). In panoramic views, glial cell size and cell density were increased in extensive areas around ventricles in POA (rostral to the hypothalamus) and PHy, both for IBA 1 and GFAP immunocytochemistry (Figures 6, 7). In our material, medial (MPA) and lateral (LPO) regions of POA were distinguished based on differences in staining. IBA 1 immunoreaction labeled both areas, but GFAP immunoreaction only labeled MPA (compare Figures 6, 7).

Inside the POA and PHy, an intense immunoreaction in microglial and astroglial cells was observed inside SFR nuclei (Figures 5, 6 dotted lines). In PHy, a high concentration of IBA1-immunoreactive cells was identified along the border of the 3^rd^ ventricle to the ventral area around and inside ARC (Figures 6A, B dotted line); however, the density of immunoreactive cells decreased in the infundibular area, allowing us to define an ARC microglial-specific region separate from the periventricular zone (Figures 6A, B).

In IBA1 immunostained sections, from PVN and the 3^rd^ ventricle, to the lateral limit of LPO, cell density decreased gradually dorsally and laterally, as shown after comparing gray density values from selected square areas in Figure 5C (mean gray values: Square 1 – 191.48/ Square 3 – 104.9). MPA and LPO limits were also well defined by the laterally and dorsally slight decrease in immunoreactive microglial cells (Figure 6).

GFAP immunoreactivity also showed a high concentration of astrocytes around the ventricle and POA, but the densest reactive areas were more restricted to the paraventricular zone (Figure 6C). As in IBA1 sections, GFAP immunostaining density decreased laterally in POA, matching MPA and LPA borders (e.g., please note these differences in cell density in details 2, 3 and 4 in Figure 7). In GFAP-immunostained sections, PVN neurons appeared as negative dots framed by densely stained astrocytic feeds (Figure 7C square 3). In the most caudal, sections immunostained against GFP show tanycytes which cross the stalk and the median eminence forming a superficial well-stained limit and a superficial barrier (Figures 7A, B squares 1 and 2).

### 3.4. Macroscopic localization of nuclei in Nissl-stained sections

**Figure 8:**
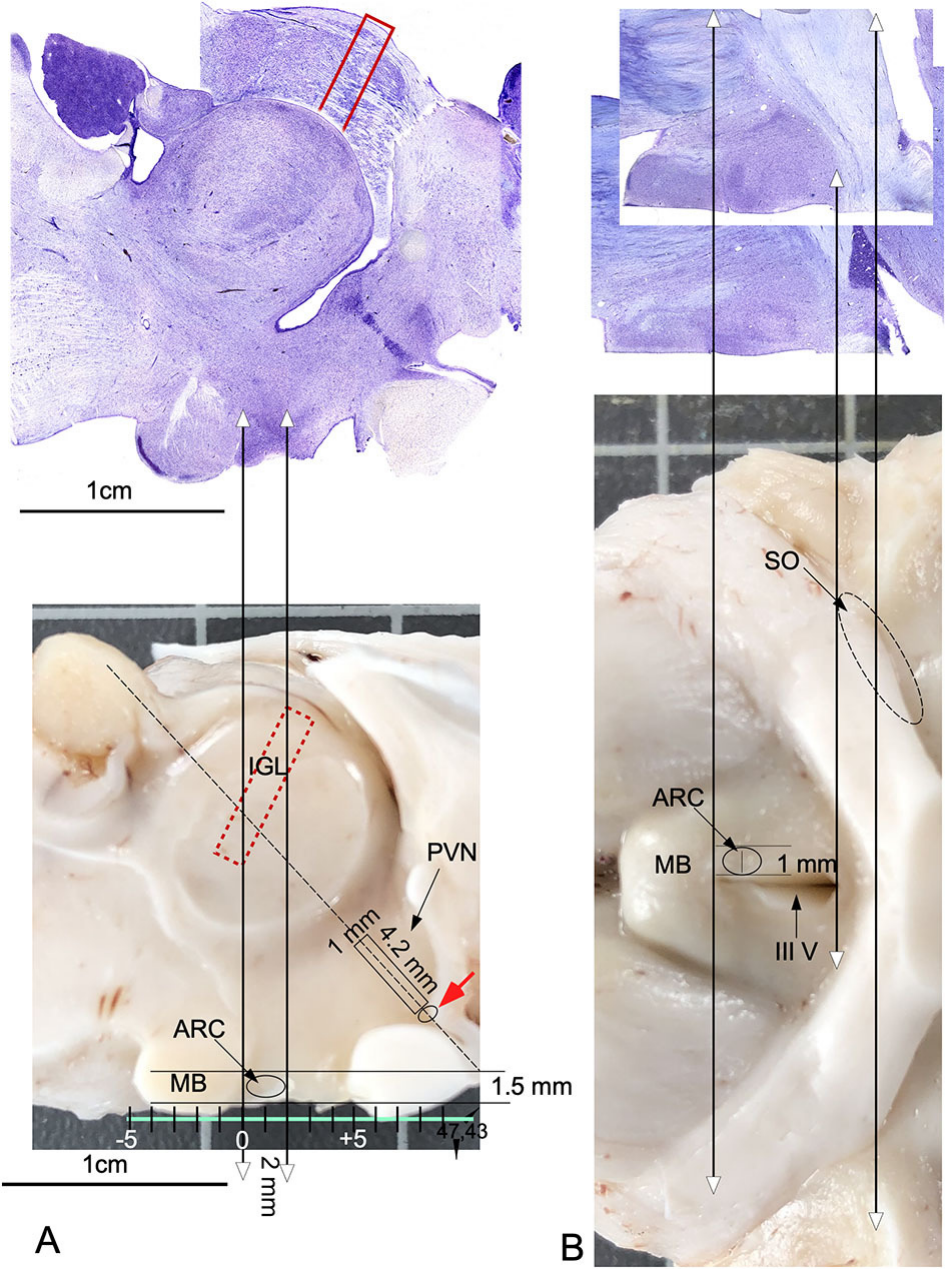
Anatomical guide for localizing nuclei on the surfaces of a sagittal midline-sectioned brain. Double headed arrows pointed the correspondence between histological sections and the macroscopic view. **(A)** Projections in a macroscopic view of coordinates measured in a sagittal Nissl section. The green line tangential to the ventral surface of the mammillary body and optic chiasma defines an optimal reference plane for calculating coordinates for precise tissue extraction. The oval line indicates the macroscopic localization of ARC. The rectangle shows the localization of PVN, and the adjacent small oval (red arrow) indicates the localization of the SCh nucleus. The red, dotted rectangle defines the estimated position of IGL within the visual thalamus. **(B)** Projections of coordinates from horizontal Nissl-stained sections transferred to a macroscopic ventral surface view of the brain. The oval circle indicates the localization and extension of ARC in a ventral view. The oval, dotted line shows the tentative localization and extension of SO and ARC. MB, Mammillary body; III V, third ventricle.

Measurements of the main central axis of the mammillary body in the three sectioning planes in histological Nissl-stained sections, compared with measures in macroscopic images, show small differences in retraction (<3%). As mentioned above, with measurements of the thickness of 20 randomly selected sections taken by three different observers (z-axis, and using a Leica X100 plan apo objective), we calculated a shrinkage of 4 (+/-) μm (final averaged thickness 36 μm). These data facilitated slight corrections in the translation of coordinates using the fixation and sectioning protocol applied here. Moreover, when coordinates are translated from frozen specimens, which is the most suitable approach to tissue extraction for molecular analysis, the measurements must be modified after evaluating changes in tissue contraction in each procedure. For micro-dissection, coordinates for extraction were defined in present paper in a caudal-to-rostral direction taking as referent point the anterior limit of the mammillary body (easily located macroscopically by its spherical shape close to the midline). From this reference point, ARC was localized in a sagittal view at 1.5 mm at the vertical axis, 2 mm at the horizontal axis (Figure 8A), and 1 mm laterally to midline (Figure 8B). By using as a reference, a parallel line to the ventral surface (tangential to the ventral most point of the mammillary body and the surface of the optic tracts – green line in Figure 8A), the periventricular region was located at its main axis oriented in an angle of 47.43^o^. By using the same reference line, SCh specimens were localized 0.7 mm dorsally to the recess (Figure 8A, red arrows), and PVN was dissected by cutting a 4.2 × 1 mm rectangle (Figure 8A rectangle). Due to its localization, sandwiched between the optic tract (OT) and the preoptic area, SO coordinates cannot be accurately defined, but a tentative localization is suggested in Figure 8B (black dotted line). Furthermore, by its convoluted structure and oblique orientation, IGL coordinates are very difficult to precisely define in macroscopic views; nevertheless, a tentative localization is also provided in Figure 8 (red dotted rectangle).

## 4. Discussion

### 4.1. General considerations

After SFR nuclei activation by changes in photoperiod, peptide regulation induces neuronal activation and plasticity (11–13) leading to morphological changes in the hypothalamus. These changes in the estrous cycle, previously studied in rats (49) and as a function of seasonal fluctuations, mainly regulated by melatonin (33) may induce size and shape differences in SFR nuclei. In this study, ewes were euthanized under specific environmental, nutritional, and estrous cycle conditions. However, potential changes in morphology may be induced by plastic reorganization after changes in hormonal induction. Therefore, the anatomical data reported here must be revised under other estral conditions.

Relative SCh, ARC and PVN differences in shape and localization were identified in this study. SO showed substantial qualitative differences in both its layout and relative size in ewes, compared with other mammals (e.g., rodents, primates. pigs) (26, 31); however, the basic neuroanatomical plan of organization of ewe SFR nuclei, is similar to that of other mammals.

In line with differences in reproductive functions between rams (e.g., mounting) and Ewes (e.g., pregnancy and lactation), strong anatomical differences have been previously shown in the nuclei studied here (sexual dimorphism) (50, 51). Accordingly, in the future a specific anatomical systematization should be performed in rams for a complete neuroanatomical description of nuclei involved in seasonality in sheep (*Ovies orientalis aries*).

KNDy neurons form an interconnected network that fires synchronously to drive GnRH release during a pulse, inducing combined differences between nuclei closely positioned in the hypothalamus (23, 24). For future molecular analysis, functional and anatomical interconnections between hypothalamic nuclei involved in seasonal sexual activation should be studied using well-defined samples of tissue restricted to specific nuclei. A study using nuclei microdissection to analyze large areas of the hypothalamus quickly immersed in liquid nitrogen reported excellent results about changes in GnRH-R mRNA levels after stress in merino sheep (52). Thus, in the present study, we provide a cytoarchitectonic guide for a more precise microdissection of SFR nuclei, enabling researchers to collect more appropriate samples for conventional proteomic or genomic techniques, individual cell sequencing or chemical analysis of hormones, transcription factors, and peptides. Preparing serial sagittal sections after micro-dissection enables to confirm the appropriate extracted areas, as previously we reported in the rat (53).

### 4.2. Intergeniculate leaflet nucleus

IGL was first described in rats (54), and golden hamsters (55) as a well-defined band of cells located between the dorsal and ventral medial geniculate bodies. When comparing rodents and higher mammals, such as primates (26) or sheep (present results), the well-defined straight band between dorsal and ventral subdivisions of the visual thalamus were masked and hidden by the complex laminar organization of the dorsal geniculate bodies. However, in our Nissl-stained sheep sections (Figure 2A), large fusiform neurons, delimiting IGL, were more clearly observed in the coronal plane (Figure 3G). In rodents, nevertheless, its architecture is well defined, but both the localization and limits are difficult to distinguish in some gyrencephalic vertebrate species (26) because primates once had a generic wide pregeniculate nucleus (PGN) included both the ventral geniculate nucleus and IGL (56). In the visual thalamus of the rock cavy (*Kerodon rupestris*; rodent), IGL is identified as a well-defined and straight band between dorsal and ventral subdivisions, but in marmosets (primates), this subdivision is masked and hidden by the complex laminar organization of the dorsal part of the LG (57). Due to its intricate architectonic organization, the primate IGL should be neuroanatomically better defined by using additional neurochemical markers (e.g., VIP immunocytochemistry). However, in the ewe, positive Cb and Cr terminal fields, most likely coming from the retina„ allowed us to trace the borders of this subdivision. In summary, due to its larger fusiform neurons and Cb and Cr immunoreactivity (Figures 5B, C), IGL can be well differentiated from the remaining visual thalamus in the ewe (Figure 3A).

### 4.3. Arcuate nucleus

Anatomically, six types of neurons have been described in previous studies using the Golgi method in the rat ARC nucleus (58). Different neurochemical phenotypes have also been reported, particularly a neuronal type in which kisspeptin, neurokinin A and dynorphin coexist, known as KNDy neurons (59). These key neurons regulate seasonality outputs for reproduction (kisspeptin, neurokinin, dynorphin) by activating GnRH pulses in sheep (32) Because these neurons are mainly located in ARC (23, 60), techniques demonstrating kisspeptin, neurokinin or dynorphin (not available in our material) should be used as complementary markers to be able to better correlate the morphology and topography of this nuclei with present results. In ARC, several chemical neuronal types have also been described; thus, an accurate analysis in the ewe will require a peptide immunocytochemistry analysis, which was not performed in our study. Nevertheless, in our material, the morphology (Nissl) and immunoreactivity (CBPs) of dorsal ARC neurons showed that they were roughly similar in size and location to KNDy neurons.

### 4.4. Paraventricular nucleus

PVN shows the highest peptidergic and glial activity in the hypothalamus and is located at the border of the third ventricle. Surrounding this structure, some regions of special interest for their association with seasonal reproduction in sheep, such as the set of A15 dopaminergic cells, regulate the negative feedback of estradiol, which in turn is another compound that regulates both GnRH production and pulses (32, 61, 62). In ewes, the columnar distribution at the sagittal plane allowed us to distinguish strongly Nissl-stained neurons into three potential subdivisions (Figure 2E).

### 4.5. Suprachiasmatic nucleus

Light cycle-sensitive pacemaker SCh neurons are directly regulated by retinal and indirectly by IGL projections through the geniculate thalamic tract (63). SCh, a highly phylogenetically conserved structure with a similar location in many species of vertebrates, is placed between the 3^rd^ ventricle and the optic chiasm and contains small and packed neurons (64). In ewes, we also observed small neurons (~430 μm^2^) and strong immunoreactivity for fibers containing Cr or Cb presumably belonging to retinal projections (Figures 5L, M).

### 4.6. Supraoptic nucleus

SO interconnects several of the seasonality nuclei (PVN and ARC), directly projecting to the pituitary gland and to the region surrounding the third ventricle (44, 45) Because SO is involved in glial activation, we will analyze its morphological features more extensively in the next section (51). Despite differences in animal size, SO nucleus and its neurons are much larger in ewes than in rodents, but they have the same topographical distribution (26, 41).

### 4.7. Glial architecture

To our best knowledge, this is the first analysis of glial cell architecture in the ewe hypothalamus and diencephalon. Glial cells are widely known to play a key role in the synthesis, recycling and delivery of hypothalamic hormones and factors (65) and thus GnRH in the hypothalamus of different mammals, including sheep (51, 66, 67). Increases in cell density and cell size around the 3^rd^ ventricle shown in our material after IBA1 and GFAP immunoreactivity, indeed expresses glial overactivation (increase cell size, number of profiles, immunostaining and cell density) which can be related to potential areas of higher metabolic regulation of sexual hormones (GnRH) (68). Accordingly, the densest areas of microglial and astroglia immunoreaction around PVN and SO, in the anterior hypothalamus, and around the ARC, in the THy, may be related with a heightened endocrine or paracrine regulation.

Notwithstanding the above, both types of reactive glial cells (as shown by larger and denser population of microgliocytes and astrocytes) are found around neurons. These findings suggest the potential role of denser immunoreactive areas with overactivated paracrine secretion. When comparing IBA1 and GFAP immunoreactivity, microglial reactivity was more extended in all section levels, suggesting a microglial more widespread in areas potentially implicated in secretory regulation (please compare Figures 6, 7). Tanycytes formed a dense ventricular barrier of the 3^rd^ ventricle in the stalk-median eminence (Figures 7A, B squares 1, 2). Due to the absence of any other reactive glial cells, this finding suggests that these areas, as in other species, regulate the access of metabolic signals to the hypothalamus (69).

### 4.8. Concluding remarks

Our results establish a guide for future histological, physiological and molecular experiments related to hypothalamic effects of interventions in farm animals aimed at improving fertility regulation (e.g., GnRH neurons, KNDy cells, and melatonin receptors). The data provided here may also have translational relevance because the sheep brain, considering its similarity in anatomy and organization, is a good experimental model for human neuroanatomical research, as recently suggested (70). Finally, by establishing a procedure for the section-to-brain translation of coordinates, this research may also help us to develop suitable procedures for nuclei/tissue extraction from specific nuclei of interest toward assessing changes in mRNA levels by RT-PCR or in protein levels by Western blot.

## Data availability statement

The raw data supporting the conclusions of this article will be made available by the authors, without undue reservation.

## Ethics statement

The animal study was reviewed and approved by the University of Salamanca.

## Author contributions

MM and CP designed the experiments. MM and IP perform the experiments. MM and RC wrote the paper. CP and JA participated in the discussion of the results and corrected the manuscript. All authors contributed to the article and approved the submitted version.

## Supplementary material

Supplementary Figure 1 Positive controls for immunocytochemistry. **(A)** Superior colliculus (SC) immunostained agains calretinin. **(B)** SC immunostained agains Calbindin and **(C)** SC immunostained agains parvalbumin. The Intermediate white layer (InWH) show immunopositive fibers for calretinin and parvalbumin but not for calbindine. Zo. zona layer, SuG superifical gray layer, Op optic nerve layer, InWH intermediate white layer, DpG Deep gray layer. **(D)** Parvalbumin immunostained of the ewe cerebellum. Note the intense immunoreactivity of basket cells and its pericellular nests, as well as positive fibers in white matter sera. ML, molecular layer, PCL, Purkinge layer, GCL, granule cell layer, WM, white matter.

Supplementary Table 1 Data summary table.
