# Supplementary figures and images for: Anatomy of hypothalamic and diencephalic nuclei involved in seasonal fertility regulation in ewes
Source: Front Vet Sci. 2023 Feb 16;10:1101024. doi: 10.3389/fvets.2023.1101024 (PMC9978410; doi:10.3389/fvets.2023.1101024)

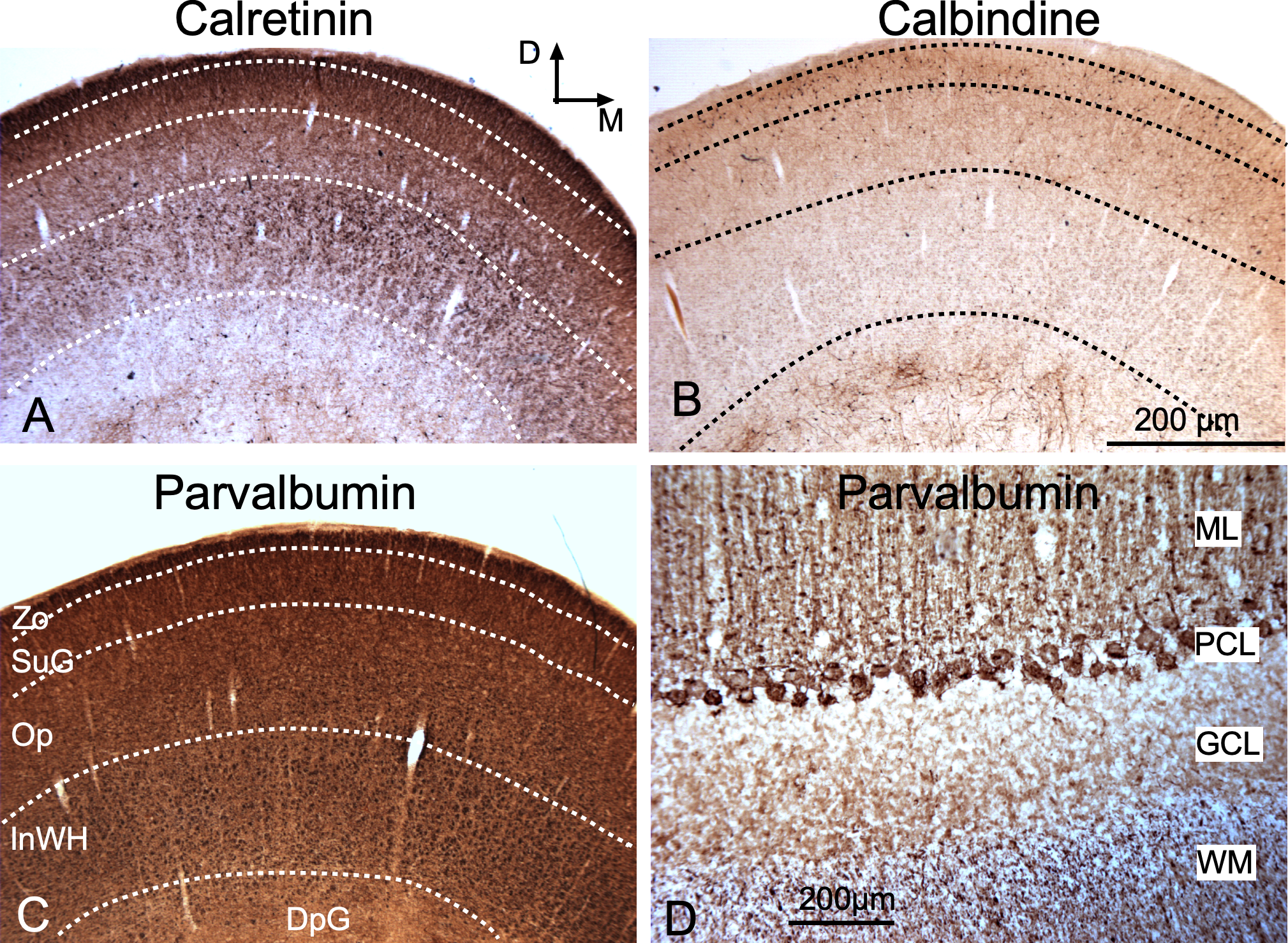

Supplement: Supplementary Figure 1 — Positive controls for immunocytochemistry. (A) Superior colliculus (SC) immunostained agains calretinin. (B) SC immunostained agains Calbindin and (C) SC immunostained agains parvalbumin. The Intermediate white layer (InWH) show immunopositive fibers for calretinin and parvalbumin but not for calbindine. Zo. zona layer, SuG superifical gray layer, Op optic nerve layer, InWH intermediate white layer, DpG Deep gray layer. (D) Parvalbumin immunostained of the ewe cerebellum. Note the intense immunoreactivity of basket cells and its pericellular nests, as well as positive fibers in white matter sera. ML, molecular layer, PCL, Purkinge layer, GCL, granule cell layer, WM, white matter. [file Image_1.TIF]

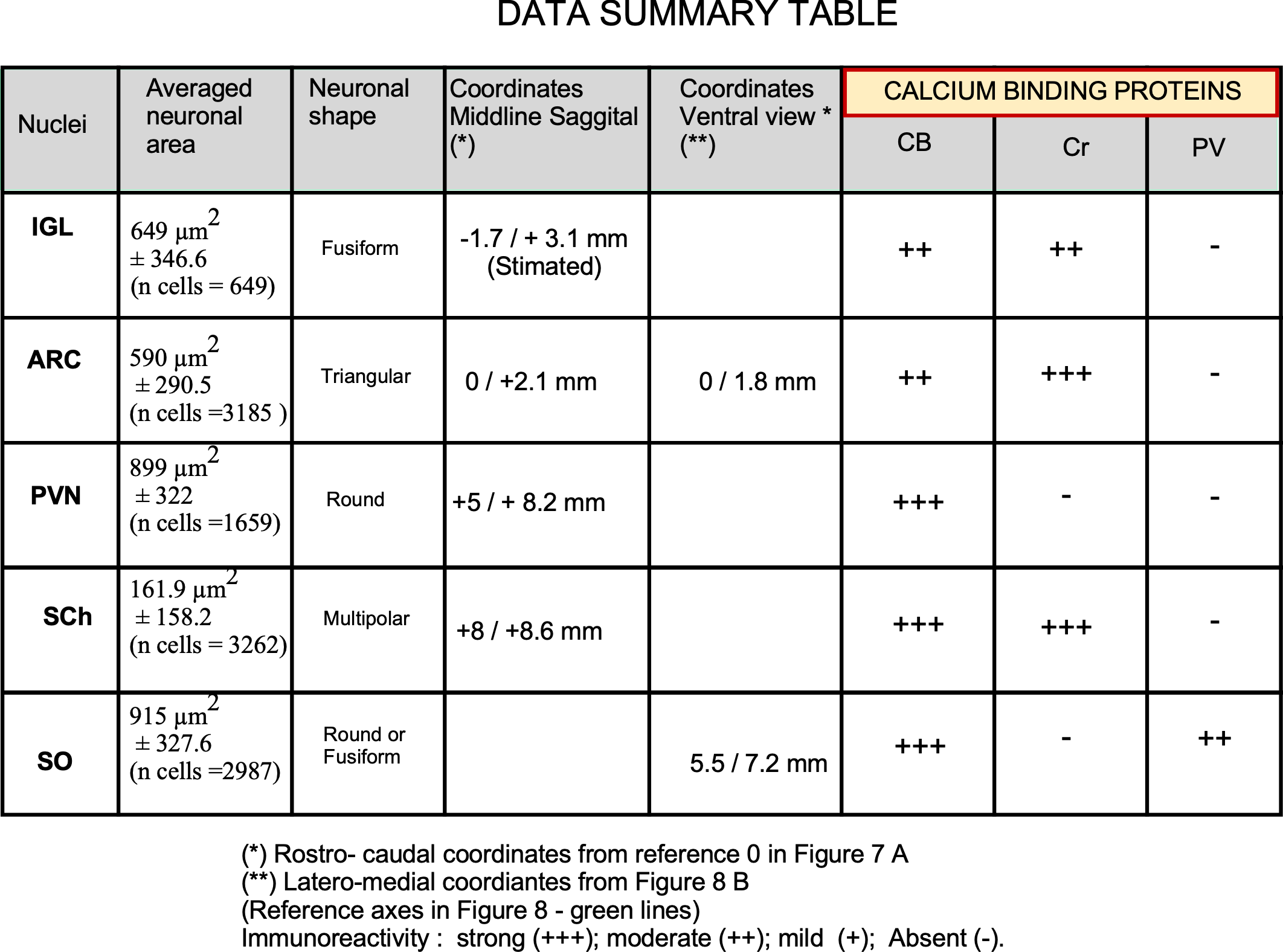

Supplement: Supplementary Table 1 — Data summary table. [file Image_2.TIF]
